# Single-Person Households: Insights from a Household Survey of Fruit and Vegetable Purchases

**DOI:** 10.3390/nu16172851

**Published:** 2024-08-26

**Authors:** Andres Silva, Maripaz Rivera, Samuel Durán-Agüero, Maria Isabel Sactic

**Affiliations:** Escuela de Nutrición y Dietética, Facultad de Ciencias para el Cuidado de la Salud, Universidad San Sebastián, Santiago 7511111, Chilemisactic@uc.cl (M.I.S.)

**Keywords:** fruit consumption, vegetable consumption, single-person households, dietary behavior, socioeconomic determinants

## Abstract

Despite the efforts made to promote consumption, some countries are not increasing their fruit and vegetable intake, while household structures are undergoing relevant changes. Fruit and vegetable consumption is necessary but not sufficient for a healthy diet. Previous research has linked adequate fruit and vegetable consumption to a lower risk of cardiovascular diseases, type 2 diabetes, and some mental health conditions. Furthermore, millions of deaths are reported annually worldwide due to diets low in fruit and vegetables, highlighting their critical public health importance. This study aims to separately analyze the purchases of fruit and vegetables in single-person households. We used three waves of the Family Budget Survey, *Encuesta de Presupuestos Familiares*, in Chile, which is nationally representative of urban areas and includes over 10,000 households in each wave. We employed descriptive statistics to examine the characteristics of the head of household and the food shopper as well as the structure, composition, and overall characteristics of households. Additionally, we performed separate analyses for fruit and vegetable purchases, using these variables to determine the marginal effect on the probability of purchasing fruit or vegetables through probit models. Results show that, from 2011–2012 to 2021–2022, the share of households not purchasing fruit and vegetables increased from 5.0% to 8.4% and that, in single-person households, it rose from 11.2% to 19.1%. Male-headed, single-person households with low education and income were more likely not to purchase fruit, and these households also have decreasing vegetable purchases. Additionally, household income significantly impacts fruit purchases but does not significantly affect vegetable purchases. Our findings highlight the importance of considering single-person households as a target population segment for future public policies to promote fruit and vegetable consumption.

## 1. Introduction

Regular intake of fruit and vegetables (FV) is associated with numerous health benefits, such as lowering the risk of cardiovascular disease and certain types of cancer [1], reducing the risk of developing type 2 diabetes mellitus [2], and lowering rates of depression in women [3]. Most countries do not meet the recommended levels of FV consumption. After conducting a systematic review of data from 162 countries, Kalmpourtzidou et al. (2020) [4] found that only 29% of the countries in Asia, 11% in Europe and Oceania, and 7% in America and Africa meet the vegetable consumption recommendation. In Latin America, for example, 19% of the population in Argentina complies with the recommendation of five FV servings per day [5], while in Chile, data from the 2016–2017 National Health Survey showed that only 15% of the population over 15 years old reached the recommendation [6]. In 2019, 2.7 million deaths worldwide were associated with a diet low in fruits, vegetables, and legumes [7], highlighting the critical importance of FV consumption. Even more, FV consumption is declining in certain countries. Comparing data from Organization for Economic Cooperation and Development (OECD) countries, it was found that, in 2021, 57% of the population reported consuming vegetables daily, compared to 59.1% in 2019 [1]. In the United States, between 1999–2000 and 2017–2018, the percentage of the population consuming at least one fruit per day decreased from 77.2% to 64.9%, while vegetable consumption did not experience a significant variation [8]. Finally, using the Family Budget Survey in Chile, Silva et al. (2021) [9] found that FV purchases decreased by 5.2% between 2011–2012 and 2016–2017.

Previous research has focused on identifying FV determinants and discussing alternatives to promote FV consumption, while FV are treated as a single type of food. Differently, our research aims to identify the population segment primarily responsible for the decrease in FV purchases. Using three waves of data from the nationally representative Family Budget Survey in urban areas of Chile, we found that single-person households are experiencing the most significant decline in FV purchases. Single-person households, where only one adult resides, provide valuable insights, as the head of household and food shopper are the same individual, offering a unique perspective on purchasing habits. This distinction is particularly relevant in our analysis, as we separated the effects of the food shopper and the head of household. In single-person households, these roles are combined, whereas in multi-adult households, the head of household and primary food shopper can be different individuals, with some households having more than one person responsible for food shopping.

Additionally, we analyzed FV separately, since we believe that their consumption is conditioned by a different set of determinants. Fruits are often favored for their sweet taste, which aligns with the instinctual preference for sweetness. In contrast, vegetables, which often contain bitter flavors, are typically less favored [10]. Also, fruits require little cooking skills to be prepared compared to vegetables, which may require more cooking skills. Moreover, the consumption of FV is associated with distinct health effects. The consumption of vegetables is associated with a lower risk of epithelial cancers, including the larynx, oral cavity, and pharynx, whereas fruit consumption is associated with a lower risk of cancer of the upper digestive tract [11]. Fruit consumption, unlike vegetable consumption, is also associated with a lower risk of metabolic syndrome [12]. Therefore, although previous research often treats fruit and vegetables as a single category, they have distinct determinants, and their consumption is associated with different health outcomes.

Given the overall importance of FV consumption for health, this article has a twofold purpose. First, we aim to separately characterize the socio-demographic aspects of the population segment that is ceasing to purchase FV, namely single-person households. Second, we intend to analyze FV purchases independently to identify the different determinants for each type of food. Additionally, this study highlights the purchasing habits of single-person households, offering valuable insights for developing plans and policies to increase FV consumption in this population segment.

## 2. Materials and Methods

We acknowledge that FV purchases are influenced by various determinants. Nevertheless, certain determinants may lead to larger effects than others. In contrast to prior studies, in this article, we want to examine the decision of choosing not to purchase FV. For this purpose, we have segmented our analysis into two parts. First, we compared a series of descriptive statistics (e.g., socio-demographics, head of household and food shopper characteristics, and quantity of fruit and vegetables purchased separately). We also compared purchased FV vs. non-purchased FV. Secondly, using a probit model, we explained the purchase decisions. These two analyses are complementary to explain the FV purchase decision.

### 2.1. Probit Analysis

Probit and logit models consist of a discrete statistical model in which the response variable has only two possible outcomes, usually represented by the values 0 and 1. The purpose of probit and logit models is to estimate the probability that an observation, with a particular set of characteristics, falls into one of two possible categories. Probit and logit models assume different distributions, while providing similar results when the outcome distribution does not have a heavy tail (as in our case). The main difference between probit and logit models, in relation to linear probability models, is that, given the nature of the data, the distribution of the function is bounded between 0 and 1 [13]. Some researchers find that logit can make interpretation easier using log odds. In our case, we chose to present marginal effects. After a probit or a logit, the marginal effect would be the same. The marginal effects represent the change in the probability of falling into one of two possible outcomes. For the study, we used a probit model to analyze the purchase decision for FV separately, since we wanted to delve into specific non-purchase determinants. We estimated the probit model, then we estimated the average marginal effects that calculate each individual observation’s marginal effect, and then we took the mean.

### 2.2. Data

This study utilizes data from three waves of the Family Budget Survey (*Encuesta de Presupuestos Familiares*, EPF for its acronym in Spanish) by the National Statistics Institute (*Instituto Nacional de Estadísticas*, INE for its acronym in Spanish) of Chile [14]. Every five years, this publicly accessible survey gathers information from more than 10,000 households across urban areas. Therefore, the data are representative of urban areas in Chile. The objective of the survey is to identify the structure of characteristics of the urban home expenditure for a year [15]. The survey has a two-stage sample design. In the initial stage, blocks are chosen based on socioeconomic and geographic stratification, followed by the random selection of households within each block. The EPF in Chile, like the Living Costs and Food Survey in the United Kingdom and the Consumer Expenditure Survey in the United States, follows international standards in terms of data collection and aggregation.

We use three consecutive data waves: EPFVII (2011–2012), EPFVIII (2016–2017), and EPFIX (2021–2022). Each wave includes data collected over 12 months (the middle of one year to the middle of the following one). Over the 12 months, for two weeks, each household needs to register their purchase in a diary. For food items, the survey includes more than 200 items. For our analysis, we consider only FV items eaten at home. For instance, we consider that a household purchases fruits if the household registers any fruit item over the two weeks that the household has kept the diary.

## 3. Results

### 3.1. Descriptive Statistics

Our analysis begins with descriptive statistics organized into two distinct sections. We present the characteristics of two separate groups of households in Table 1 and Table 2: those that do not purchase fruits and those that do not purchase vegetables, respectively. This segmentation allows us to explore and highlight the differing characteristics of households purchasing only one type of food—either fruit or vegetables. Our focus extends to several key areas: the quantity of fruit or vegetable servings purchased, various household characteristics, details about the primary food shopper, and the overall structure and composition of the household.

Table 1 presents the statistics for households that report purchasing vegetables but no fruits. First, we found that 19.5% of households report not buying fruits, and these households, despite not buying fruits, purchase on average 0.6 portions of vegetables. In contrast, households that report buying fruits purchase an average of 2.5 portions of vegetables, which is four times the amount bought by the group that does not buy fruits. Furthermore, heads of household in fruit-purchasing households are older, but no significant differences were found between the means for the gender of the head of household or education level. Regarding the characteristics of the food shopper, they are also significantly older in fruit-purchasing households compared to non-fruit purchasers. Additionally, there is a higher share of women responsible for shopping in both groups. However, in the group that buys fruits, the second-largest share consists of a combination of genders, meaning more than one person is responsible for household shopping. In contrast, in households that report buying fruits, the share of male food shoppers is lower compared to those that do not buy fruits.

Regarding household composition, we observe that households that do not purchase fruits have, on average, fewer men, women, and children, suggesting they are smaller households. In terms of structure, the group with the largest share of non-fruit purchasers consists of households with adults without children, followed by single-person households. These proportions change in households that purchase fruits; although households with adults and without children still represent the largest share, they are now followed by households with multiple adults and children. This difference in proportion shows that, on average, there are more single-person households that do not purchase fruits, with a 17.0 percentage point difference between groups. Finally, we observe that only 18.2% of households that do not purchase fruits have a high income, compared to 23.5% in the group that does purchase fruits.

Table 2 presents a similar analysis to the previous table, but now showing the characteristics for vegetable purchaser households and non-vegetable purchaser households. For this analysis, we found that 14.6% of households report not buying vegetables. These households, despite not purchasing vegetables, report buying 0.45 portions of fruits, which is significantly lower than the group that does report buying vegetables, who purchase an average of 2.10 portions of fruits—nearly five times more than the non-vegetable purchasers.

Comparing head of household characteristics, vegetable purchasers are on average significantly older, with no differences observed in gender or education level. Regarding the characteristics of the food shopper, these follow the same trends reported for households that buy and do not buy fruits. The primary shoppers are significantly older on average and, similar to the previous findings, women are primarily responsible for shopping, followed by a combination of genders in households that buy vegetables. In households that do not buy vegetables, men are the second-largest group responsible for shopping, with nearly 20% more men handling shopping compared to households that do buy vegetables. Similarly, there is no clear trend regarding the relationship between vegetable purchases and education level for the food shopper.

Household composition and structure follow the same trends as in the previous analysis. On average, households that do not purchase vegetables are composed of fewer men, women, and children. In terms of structure, for households that do not buy vegetables, the group with the largest share consists of single-person households, followed by households with adults without children. Conversely, households that purchase vegetables have a higher proportion of households with one adult without children, followed by households with multiple adults and children. In this analysis, the proportion of single-person households that do not buy vegetables is nearly 20 percentage points higher than those that do buy vegetables, which is slightly higher than what was reported for fruit purchases. Finally, we observe that households that do not buy vegetables have, on average, lower incomes compared to those that do, similar to what was observed in the analysis for fruit purchases.

Using data from 2011–2012, 2016–2017, and 2021–2022, Figure 1 shows how non-fruit purchaser households and non-vegetable purchaser households have increased over a decade. As presented in Table 1 and Table 2, households that do not purchase one can still purchase the other one; therefore, the share of households that do not purchase FV is likely to be smaller than the share that does not purchase one of them. In the overall sample, 5.0% did not purchase FV in 2011–2012, 8.1% in 2016–2017, and 8.4% in 2021–2022. Regarding the overall purchased portions of FV, we found 4.1 portions in 2011–2012, 3.9 portions in 2016–2017, and 4.0 portions in 2021–2022. Specifically, in the single-person household sub-sample, 11.2% did not purchase FV in 2011–2012, 17.9% in 2016–2017, and 19.1% in 2021–2022, while there were FV purchases of 6.3 portions in 2011–2012, 5.2 portions in 2016–2017, and 4.8 portions in 2021–2022. Therefore, despite all the public effort to promote FV consumption, it is far from the recommended five portions of FV and has decreased over a decade. Additionally, single-person households are leading the decreasing trend (in terms of the proportion of non-FV purchasers and FV portions).

### 3.2. Probit Results

Finally, we conducted a set of probit models. Table 3 presents the marginal effects after a probit analysis, where the dependent variable is whether or not to purchase fruit. The marginal effects indicate the change in the probability of purchasing fruit resulting from a change in the independent variable. The overall results show that head of household characteristics are not significant, except for the gender of the head of household, as households with a female head are less likely to purchase fruits compared to their male counterparts. On the other hand, food shopper characteristics significantly affect the probability of purchasing fruit. Specifically, having a female food shopper or more than one food shopper is associated with an increased probability of purchasing fruit. Additionally, higher education levels of the food shopper and higher household income are linked to an increased probability of purchasing fruit. These results are consistent across the three waves of the survey. Furthermore, household composition significantly affects the probability of purchasing fruit for the second and third survey waves, while no significant effect was observed in the first wave. Finally, household structure also has significant effects on the probability of purchasing fruits. Compared to single-person households (omitted variable), all other household structures have a higher probability of purchasing fruits, with particularly larger marginal effects observed when there is more than one adult in the household. Lastly, high-income households have significant positive marginal effects on fruit purchases on the first and second wave of the survey.

**Figure 2:**
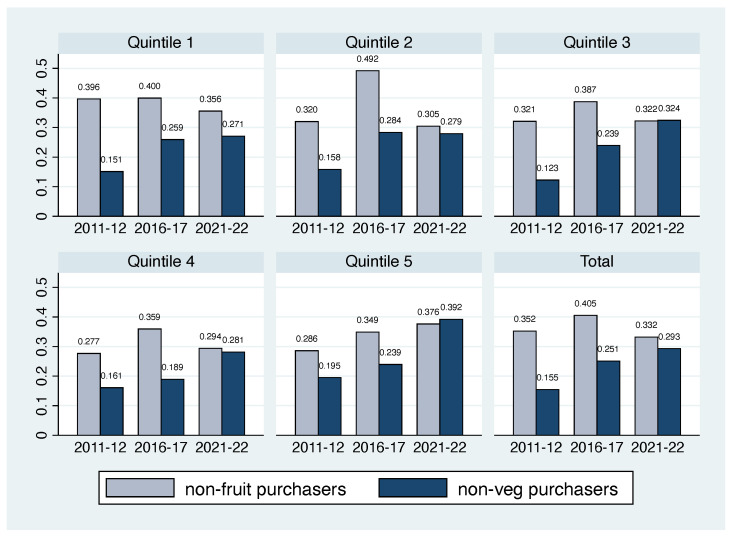
Non-purchasers by household income quintiles—Single-person households. The data presented in the figures use statistical survey weights. The figure only uses single-person households. The non-fruit purchase column, presented in light blue, corresponds to the percentage of the population that does not purchase fruits. In a similar way, the non-vegetable purchase column is presented in dark blue.

Table 4 presents the marginal effects obtained from the probit model using the vegetable purchase decision as the dependent variable. Similarly to Table 3, head of household characteristics are not significant. Regarding food shopper characteristics, female food shoppers or a combination of genders as food shoppers have positive significant marginal effects on vegetable purchases, while none of the education categories shows significant marginal effects for the first and third waves. Only the higher education categories in the second wave present significant effects. Similarly, household composition shows comparable results for FV purchases. In the first wave, the only significant variable is the number of children, while in the second and third waves, all household compositions exhibit significant marginal effects. Consistent with Figure 2, household structure plays a crucial role in explaining vegetable purchasing patterns. Specifically, single-person households (the reference category) have the lowest probability of purchasing vegetables, whereas the other three household categories display positive and significant marginal effects. In contrast to fruit purchases, high-income households do not present significant marginal effects on vegetable purchases.

In summary, Table 3 shows that food shopper characteristics, household structure, and household income are significant determinants of fruit purchase decisions. In the case of vegetables, Table 4 indicates that the gender of the food shopper and household structure are significant determinants of vegetable purchase decisions. Additionally, contrary to what was found for fruit purchases, household income does not appear to be a significant determinant for vegetable purchases. Therefore, FV purchase decisions are influenced by different sets of determinants. Single-person households and those with a male food shopper are identified as vulnerable population segments for both FV.

## 4. Discussion

Our main finding is that single-person households are driving the trend of not purchasing fruit and vegetables (FV). From 2011–2012 to 2021–2022, the proportion of households not buying FV increased from 5.0% to 8.4%, with single-person households experiencing a notable rise from 11.2% to 19.1%. Additionally, we found that FV purchases are influenced by different factors, indicating that they should be analyzed separately. For instance, our results show that household income significantly affects fruit purchases but not vegetable purchases, which may be related to differences in the prices of these products. Therefore, given the importance of household structure in analyzing FV purchases and the significant changes observed in single-person households, we focused our analysis on the determinants of FV purchases specifically within this group. This focus is particularly relevant for formulating targeted policies to increase FV purchases among these households and help them benefit from the associated health advantages.

In recent decades, there has been a rapid increase in the prevalence of single-person households in Latin America [16]. This trend is also observed in Chile, where the Family Budget Survey for 2021–2022 reported a prevalence of 16.6% of single-person households in urban areas, representing an increase of 3.5 percentage points compared to what was reported for 2016–2017. A relevant portion of previous research on single-person households comes from South Korea. Using data from home meal replacement, Kim et al. (2021) [17] identified three types of single-person households: (i) Utilitarians are households whose members are in their 20s, unmarried, and unemployed, with relatively lower income. For this group, convenience and economic value are the key food choice determinants. (ii) Health-conscious utilitarians are households in their 40s and 50s, in which quality is also important in their food choices. (iii) Variety-seekers are well educated households. In their food choices, they add trendiness as a relevant determinant. In addition, being a single-person household is associated with purchasing delivery/take-out foods, eating out, skipping breakfast [18], and cooking less frequently [19]. Finally, complementing research beyond South Korea, during the COVID-19 pandemic, using data from three European countries, Janssen et al. (2021) [20] found that single-person households were less likely to change their food consumption patterns. Based on experiments in the UK, Kawai et al. (2021) [21] found that people stated that food tasted better and that they ate more compared to eating alone. It is not clear why people tend to eat more in a social context. It may be related, especially among the elderly, to the fact that a high frequency of eating alone is associated with depression [22] and loss of appetite [23].

In addition, we analyzed the effects of head of household vs. food shopper and gender with respect to FV purchase decisions. Previous evidence consistently found that male food shoppers eat fewer FV than female food shoppers [24,25]. Along the same line, compared to female-headed households, Terin et al. (2019) [26] found that male-headed households are 2.4% less likely to purchase FV, while Ogundari (2013) [27] found that male-headed households are associated with an overall decrease in FV expenditures at home. Verain et al. (2020) [28] found that men, more than women, are more likely to not consume FV. Rodrigues et al. (2020) [29] explain that stereotypes are deeply embedded in food-related gender roles, which show that cooking is presented as an everyday domestic task for women. Even in developed countries like the UK, mothers remain as the source of cooking skills, and they are largely the ones in charge of cooking at home [30].

Our results are able to disentangle the gender effect by type of food. For fruit, the male-headed household is associated with an increase in the purchasing probability. However, for vegetables, the gender of the head of household is not a significant determinant. For FV, a male food shopper is associated with a smaller probability of purchasing. The aforementioned is consistent with the study of Lee and Allen (2021) [31], who found that men are more prone to practice negative eating habits compared to women. The FV gender disparity also may be linked with cooking skills. Using the Health and Lifestyles Survey in the UK, Caraher et al. (1999) [32] found that 23% of the surveyed men stated that they did not cook or did not feel confident cooking from basic ingredients, compared to 6% of women. Therefore, men, more than women, can be a target of public policies, especially when they are in charge of food shopping.

Household income has been pointed out as one of the main determinants of FV consumption. Some previous research has even estimated that the price of healthy food is a proxy for economic barriers for a healthy diet. Previous research has shown that populations from a low socioeconomic level consume fewer FV compared to populations from a high socioeconomic level [33]. However, most of this previous research analyzes FV together, while few articles analyze fruit and vegetables separately. We found that household income is a significant determinant only for fruit purchases, while it is not linked with a significant effect for vegetable purchases. However, the fact that income is not linked to vegetable consumption/purchases has been mentioned before [34]. According to Mora (2022) [35], in Mexico, household income and parents’ educational level explain FV consumption. Using data from 18 countries, Miller et al. (2016) [36] found that a portion of vegetables is cheaper than a portion of fruit, while low-income countries have the lowest prices on vegetables and the highest prices on fruit. It could be the case that vegetable consumption may be led by other determinants, such as cooking skills [37], instead of being directly related to income. In this sense, more research is needed that is able to distinguish the significant determinants for fruit and vegetables separately.

Finally, previous research has shown that education plays a relevant role in explaining FV consumption. Likewise, lower maternal education has been related to lower consumption of FV in childhood [38]. According to McKinnon et al. (2014) [39], education can help reduce diet differences among socioeconomic segments. Recognizing that education for all household members is preferable, our results show that education is associated with an increase in fruit purchases rather than in vegetable purchases. Food shopper education is associated with an increase in the probability of purchasing fruit, while no significant effect is associated with head of household education.

We need to approach these findings with caution, since the available data only consider home purchases, not actual consumption. Not all purchased food is ultimately consumed; some portions are inedible, and some food becomes waste. Additionally, the data do not account for food consumed away from home, such as in restaurants, which may explain a relevant portion of the purchases, especially by high-income households. Previous evidence indicates that single-person households have distinctive food consumption habits. For instance, Pachucki et al. (2018) [40] found that unaccompanied meals (single-person) and a higher frequency of meals away from home are associated with poorer diet quality.

**Figure A1:**
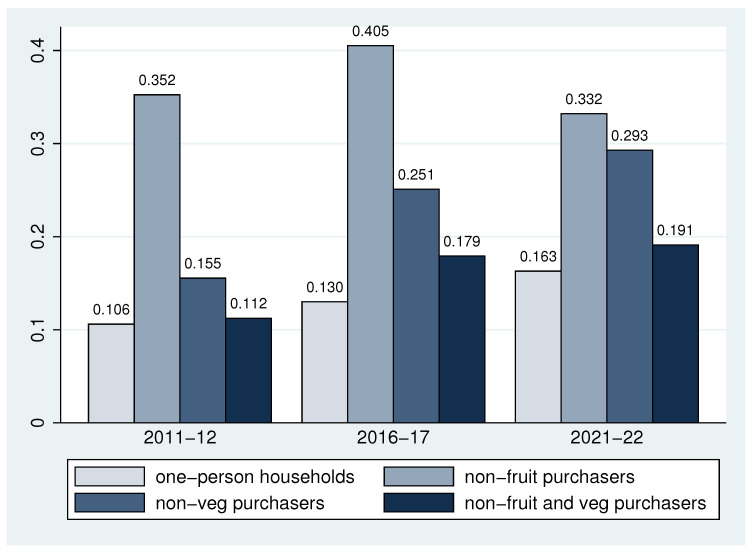
Single-person households and share of non-purchase of fruit and vegetables. The data presented in the figures use statistical survey weights. Bars represent the share per survey wave of each variable.

In summary, our findings indicate that single-person households represent the largest proportion of non-purchasers of FV, both individually and collectively (see Figure A1). Additionally, as household income increases, there is an expected rise in the prevalence of single-person households within the population. As this demographic continues to grow, understanding the factors influencing their FV purchases and consumption becomes crucial. Gaining insight into these determinants is vital for crafting targeted public policies aimed at enhancing the intake of these essential food groups, thereby improving individual health benefits. Additionally, such policies could alleviate the strain on public health systems by potentially reducing the prevalence of diseases associated with insufficient FV consumption.

Our findings suggest that public policies associated with the purchase and consumption of FV should differentiate between these two food groups and consider social variables such as the gender of the shopper. This recommendation stems from our observation that households with male food shoppers are less likely to buy FV. Consequently, it is crucial that these policies also investigate other gender-related factors that might influence FV purchasing and consumption behaviors. These factors include culinary skills related to selection and preparation, as well as preferences or aversions to specific types of products, flavors, or textures. A comprehensive understanding of these influences is essential for formulating more effective public policies.

Additionally, we identified the need for future research on the actual consumption of FV within households. While the survey used for this analysis provides relevant information on household composition and purchasing habits, it lacks detailed data on the consumption patterns of these products within the home. This represents a significant limitation of the study, as it only analyzes purchases and not actual consumption. Furthermore, another important limitation to consider is that the study only accounts for FV purchased for consumption and preparation within the home, excluding those bought outside, such as in prepared meals at restaurants.

## 5. Conclusions

Given all the public efforts, it may be surprising that some countries, such as Chile, are not increasing FV purchases. After analyzing data over a decade of the Family Budget Survey, which is representative of the main urban centers, we found that single-person households, especially male-headed ones, are more likely to not purchase FV, both together and separately. Our results show that single-person households are a growing segment of the population. Considering their purchasing habits, the results suggest that greater attention should be paid to focusing policies on increasing the purchase and consumption of FV in this population. Therefore, we expect that this research provides evidence to inform public policies directed at single-person households.

## Figures and Tables

**Figure 1:**
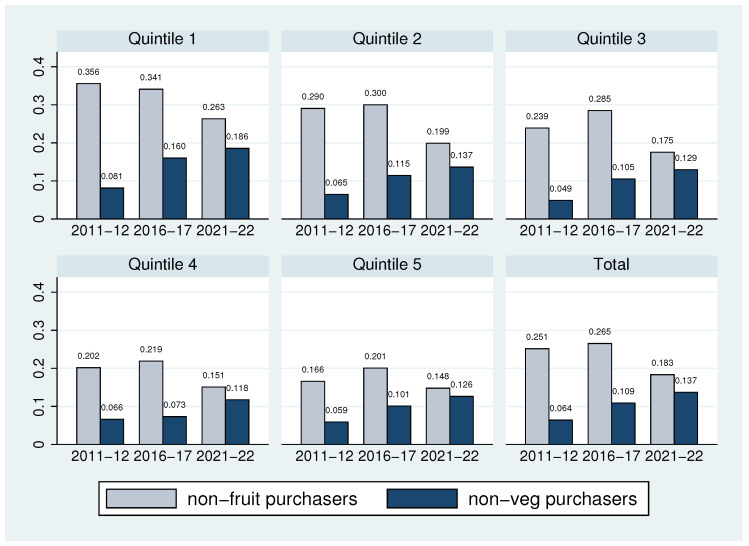
Non-purchasers by household income quintiles—Overall. The data presented in the figures use statistical survey weights. The figure uses the full sample. The non-fruit purchase column, presented in light blue, corresponds to the percentage of the population that does not purchase fruits. In a similar way, the non-vegetable purchase column is presented in dark blue.

**Table 1:** Descriptive statistics for fruit purchases.

	Non-Fruit Purchasers	Fruit Purchasers	Difference
	Mean	Mean	
Fruit portions	0.000	2.295	−2.218 ***
Vegetable portions	0.636	2.488	−1.806 ***
Head of Household Characteristics			
Gender of head, 1 = male, 2 = female	1.428	1.429	0.017
Age of head of household, years	48.173	50.628	−1.699 ***
Education, less than 4 years	0.045	0.034	0.015 ***
Education, 4–8 years	0.085	0.076	0.018 **
Education, 8–12 years	0.146	0.154	0.005
Education, 12–16 years	0.444	0.456	−0.017
Education, more than 16 years	0.280	0.281	−0.020 *
Food Shopper Characteristics			
Gender of food shopper, male	0.292	0.157	0.105 ***
Gender of food shopper, female	0.424	0.445	−0.006
Gender of food shopper, combination	0.284	0.398	−0.099 ***
Age of food shopper, years	47.339	49.668	−1.484 ***
Education, less than 4 years	0.040	0.023	0.018 ***
Education, 4–8 years	0.086	0.077	0.019 **
Education, 8–12 years	0.164	0.199	−0.012
Education, 12–16 years	0.466	0.462	−0.015
Education, more than 16 years	0.245	0.239	−0.010
Household Composition			
Number of men	1.221	1.573	−0.304 ***
Number of women	1.308	1.488	−0.166 ***
Number of children	0.583	0.700	−0.106 ***
Household Structure			
Single-person	0.295	0.133	0.137 ***
Adults/seniors without children	0.345	0.434	−0.068 ***
An adult/senior with children	0.075	0.050	0.023 ***
Adults/seniors with children	0.285	0.382	−0.092 ***
Household Characteristics			
High income	0.182	0.235	−0.054 ***
Observations	2926	12,085	15,011

A portion of FV corresponds to 80 grams. Statistics based on data from (2021–2022) and representative weights of main urban areas at the national level. By household structure, “adults/seniors without children” corresponds to households with more than one person over 18 years old and no children, while “adult/senior with children” corresponds to households with only an adult and children. * *p* < 0.1, ** *p* < 0.05, *** *p* < 0.01.

**Table 2:** Descriptive statistics for vegetable purchases.

	Non-Veg Purchasers	Vegetable Purchasers	Difference
	Mean	Mean	
Vegetable portions	0.000	2.490	−2.476 ***
Fruit portions	0.450	2.101	−1.575 ***
Head of Household Characteristics			
Gender of head, 1 = male, 2 = female	1.413	1.431	−0.003
Age of head of household, years	47.609	50.586	−2.350 ***
Education, less than 4 years	0.032	0.036	−0.001
Education, 4–8 years	0.058	0.081	−0.018 **
Education, 8–12 years	0.132	0.156	−0.012
Education, 12–16 years	0.429	0.457	−0.020
Education, more than 16 years	0.348	0.270	0.051 ***
Food Shopper Characteristics			
Gender of food shopper, male	0.344	0.156	0.149 ***
Gender of food shopper, female	0.408	0.446	−0.029 *
Gender of food shopper, combination	0.247	0.398	−0.120 ***
Age of food shopper, years	47.072	49.585	−1.831 ***
Education, less than 4 years	0.030	0.025	0.007
Education, 4–8 years	0.063	0.081	−0.012
Education, 8–12 years	0.143	0.200	−0.036 ***
Education, 12–16 years	0.447	0.465	−0.019
Education, more than 16 years	0.317	0.228	0.060 ***
Household Composition			
Number of men	1.125	1.569	−0.371 ***
Number of women	1.243	1.489	−0.207 ***
Number of children	0.528	0.703	−0.134 ***
Household Structure			
Single-person	0.348	0.134	0.179 ***
Adults/seniors without children	0.314	0.434	−0.098 ***
An adult/senior with children	0.073	0.052	0.022 ***
Adults/seniors with children	0.265	0.380	−0.102 ***
Household Characteristics			
High income	0.208	0.228	−0.022 *
Observations	2194	12,817	15,011

A portion of FV corresponds to 80 grams. Statistics based on data from EPFIX (2021–2022) and representative weights of main urban areas at the national level. By household structure, “adults/seniors without children” corresponds to households with more than one person over 18 years old and no children, while “adult/senior with children” corresponds to households with only an adult and children. * *p* < 0.1, ** *p* < 0.05, *** *p* < 0.01.

**Table 3:** Fruit purchase marginal effects after probit estimation.

Variable	EPFVII (2011–2012)		EPFVIII (2016–2017)		EPFIX (2021–2022)	
	Effect	SD	Effect	SD	Effect	SD
Head of Household Characteristics						
Gender of head, 1 = male, 2 = female	−0.027 **	0.010	−0.025 **	0.009	−0.023 **	0.009
Age of head of household, years	0.001	0.001	0.002 ***	0.001	0.001	0.001
Education, 4–8 years	−0.043	0.029	0.030	0.026	−0.005	0.026
Education, 8–12 years	0.008	0.028	0.040	0.027	0.008	0.027
Education, 12–16 years	0.010	0.028	0.042	0.029	0.035	0.028
Education, more than 16 years	0.036	0.033	0.041	0.033	0.041	0.031
Food Shopper Characteristics						
Gender of food shopper, female	0.046 ***	0.013	0.054 ***	0.012	0.068 ***	0.013
Gender of food shopper, combination	0.043 **	0.016	0.071 ***	0.012	0.071 ***	0.012
Age of food shopper	0.004 ***	0.001	0.002 **	0.001	0.002 **	0.001
Education, 4–8 years	0.077 *	0.035	−0.008	0.030	0.041	0.030
Education, 8–12 years	0.092 **	0.034	0.037	0.031	0.077 *	0.032
Education, 12–16 years	0.138 ***	0.035	0.066 *	0.032	0.075 *	0.034
Education, more than 16 years	0.181 ***	0.039	0.135 ***	0.035	0.093 *	0.037
Household Composition						
Number of men	0.002	0.006	0.045 ***	0.005	0.034 ***	0.005
Number of women	−0.002	0.006	0.020 ***	0.005	0.019 ***	0.005
Number of children	0.015	0.008	−0.038 ***	0.008	−0.028 ***	0.008
Household Structure						
Adults/seniors without children	0.126 ***	0.019	0.086 ***	0.015	0.080 ***	0.014
An adult/senior with children	0.073 **	0.028	0.101 ***	0.022	0.076 ***	0.019
Adults/seniors with children	0.157 ***	0.023	0.125 ***	0.018	0.107 ***	0.018
Household Characteristics						
High income	0.045 ***	0.013	0.040 ***	0.011	0.008	0.010
Observations	10,431		15,183		15,009	

EPF corresponds to the abbreviation of the survey name in Spanish (Encuesta de Presupuestos Familiares), as detailed in previous sections. Roman numerals VII, VIII, and IX denote the respective survey waves. Marginal effects are computed after probit estimation. We used the “margins” post-estimation command in Stata BE 17 to estimate the average marginal effect. Statistical significance levels are indicated as follows: * *p* < 0.1, ** *p* < 0.05, *** *p* < 0.01.

**Table 4:** Vegetable purchase marginal effects after probit estimation.

Variable	EPFVII (2011–2012)		EPFVIII (2016–2017)		EPFIX (2021–2022)	
	Effect	SD	Effect	SD	Effect	SD
Head of Household Characteristics						
Gender of head, 1 = male, 2 = female	0.002	0.006	−0.003	0.007	−0.015	0.008
Age of head of household, years	0.000	0.000	0.000	0.000	0.001 *	0.000
Education, 4–8 years	−0.024	0.017	−0.018	0.016	0.010	0.021
Education, 8–12 years	−0.017	0.016	−0.013	0.017	−0.023	0.023
Education, 12–16 years	−0.007	0.015	−0.038 *	0.018	−0.013	0.024
Education, more than 16 years	−0.005	0.018	−0.033	0.021	−0.021	0.027
Food Shopper Characteristics						
Gender of food shopper, female	0.024 **	0.008	0.043 ***	0.009	0.083 ***	0.013
Gender of food shopper, combination	0.031 ***	0.009	0.046 ***	0.009	0.090 ***	0.012
Age of food shopper	0.001 **	0.000	0.001**	0.000	0.001	0.000
Education, 4–8 years	0.020	0.021	0.025	0.027	0.023	0.029
Education, 8–12 years	0.010	0.021	0.047	0.028	0.053	0.030
Education, 12–16 years	0.014	0.021	0.065 *	0.029	0.048	0.032
Education, more than 16 years	0.022	0.023	0.071 *	0.031	0.038	0.034
Household Composition						
Number of men	−0.003	0.003	0.031 ***	0.004	0.028 ***	0.005
Number of women	−0.003	0.003	0.018 ***	0.004	0.020 ***	0.005
Number of children	0.016 **	0.005	−0.018 **	0.006	−0.024 ***	0.007
Household Structure						
Adults/seniors without children	0.099 ***	0.015	0.085 ***	0.012	0.079 ***	0.013
An adult/senior with children	0.067 **	0.021	0.060 ***	0.017	0.071 ***	0.017
Adults/seniors with children	0.104 ***	0.018	0.100 ***	0.015	0.090 ***	0.016
Household Characteristics						
High income	−0.005	0.007	−0.013	0.007	−0.002	0.008
Observations	10,431		15,183		15,009	

EPF corresponds to the abbreviation of the survey name in Spanish (Encuesta de Presupuestos Familiares), as detailed in previous sections. Roman numerals VII, VIII, and IX denote the respective survey waves. Marginal effects are computed following probit estimation. We used the “margins” post-estimation command in Stata BE 17 to estimate the average marginal effect. Statistical significance levels are indicated as follows: * *p* < 0.1, ** *p* < 0.05, *** *p* < 0.01.

**Figure A2:**
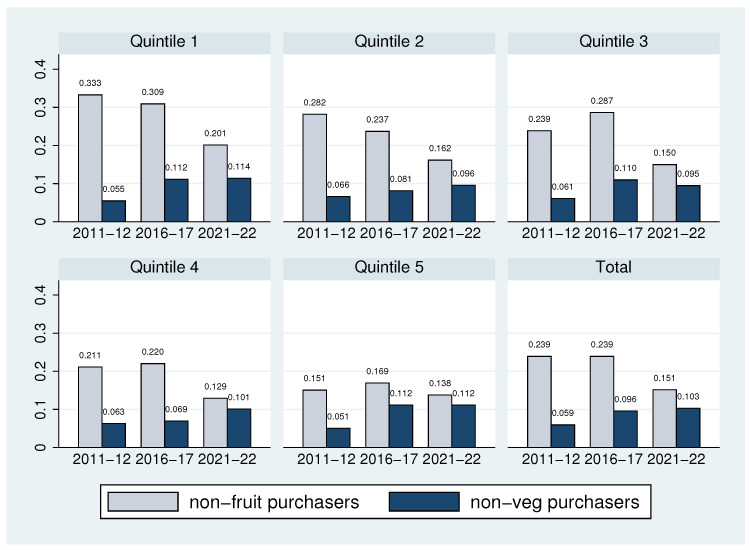
Non-purchasers by quintile and structure—Adults/seniors without children. The data presented in the figures use statistical survey weights. The figure uses only households with adults/seniors without children. The non-fruit purchase column, presented in light blue, corresponds to the percentage of the population that does not purchase fruits. In a similar way, the non-vegetable purchase column is presented in dark blue.

**Figure A3:**
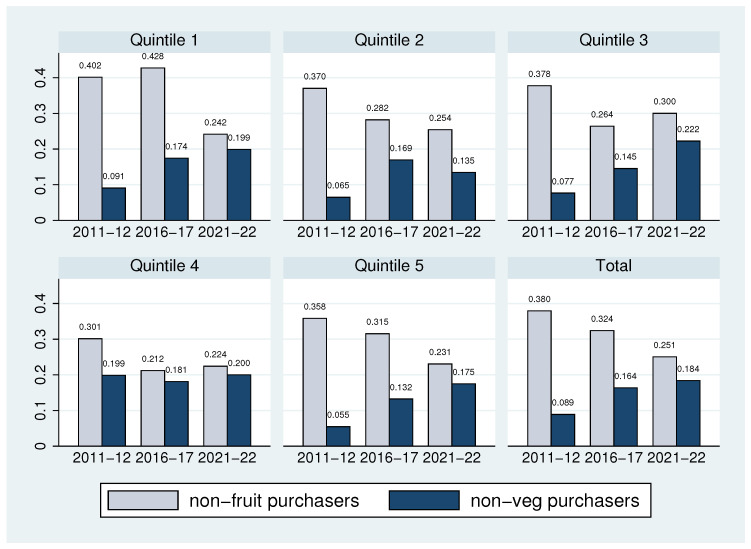
Non-purchasers by quintile and structure—Adult/senior with children. The data presented in the figures use statistical survey weights. The figure uses only households with one adult/senior with children. The non-fruit purchase column, presented in light blue, corresponds to the percentage of the population that does not purchase fruits. In a similar way, the non-vegetable purchase column is presented in dark blue.

**Figure A4:**
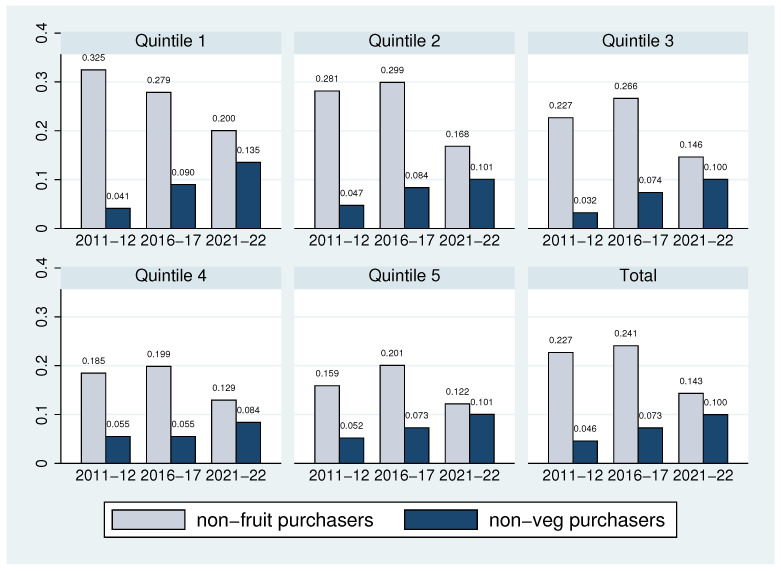
Non-purchasers by quintile and structure—Adults/seniors with children. The data presented in the figures use statistical survey weights. The figure uses only households with adults/seniors with children. The non-fruit purchase column, presented in light blue, corresponds to the percentage of the population that does not purchase fruits. In a similar way, the non-vegetable purchase column is presented in dark blue.

## Data Availability

The original data presented in the study are openly available at https://www.ine.gob.cl/estadisticas/sociales/ingresos-y-gastos/encuesta-de-presupuestos-familiares.

## Appendix A

**Table A1:** Full table of descriptive statistics for fruit purchases.

	Non-Fruit Purchasers		Fruit Purchasers		Difference
	Mean	SD	Mean	SD	
Fruit portions	0.000	(0.000)	2.295	(2.260)	−2.218 ***
Vegetable portions	0.636	(1.286)	2.488	(2.382)	−1.806 ***
Head of Household Characteristics					
Gender of head, 1 = male, 2 = female	1.428	(0.495)	1.429	(0.495)	0.017
Age of head of household, years	48.173	(16.041)	50.628	(15.348)	−1.699 ***
Education, less than 4 years	0.045	(0.208)	0.034	(0.180)	0.015 ***
Education, 4–8 years	0.085	(0.279)	0.076	(0.265)	0.018 **
Education, 8–12 years	0.146	(0.353)	0.154	(0.361)	0.005
Education, 12–16 years	0.444	(0.497)	0.456	(0.498)	−0.017
Education, more than 16 years	0.280	(0.449)	0.281	(0.449)	−0.020 *
Food Shopper Characteristics					
Gender of food shopper, male	0.292	(0.455)	0.157	(0.364)	0.105 ***
Gender of food shopper, female	0.424	(0.494)	0.445	(0.497)	−0.006
Gender of food shopper, combination	0.284	(0.451)	0.398	(0.489)	−0.099 ***
Age of food shopper, years	47.339	(15.510)	49.668	(14.353)	−1.484 ***
Education, less than 4 years	0.040	(0.195)	0.023	(0.150)	0.018 ***
Education, 4–8 years	0.086	(0.280)	0.077	(0.267)	0.019 **
Education, 8–12 years	0.164	(0.370)	0.199	(0.399)	−0.012
Education, 12–16 years	0.466	(0.499)	0.462	(0.499)	−0.015
Education, more than 16 years	0.245	(0.430)	0.239	(0.427)	−0.010
Household Composition					
Number of men	1.221	(1.001)	1.573	(1.031)	−0.304 ***
Number of women	1.308	(0.934)	1.488	(0.997)	−0.166 ***
Number of children	0.583	(0.906)	0.700	(0.959)	−0.106 ***
Household Structure					
Single-person	0.295	(0.456)	0.133	(0.340)	0.137 ***
Adults/seniors without children	0.345	(0.475)	0.434	(0.496)	−0.068 ***
An adult/senior with children	0.075	(0.263)	0.050	(0.218)	0.023 ***
Adults/seniors with children	0.285	(0.451)	0.382	(0.486)	−0.092 ***
Household Characteristics					
High income	0.182	(0.386)	0.235	(0.424)	−0.054 ***
Observations	2926		12,085		15,011

A portion of FV corresponds to 80 grams. Statistics based on data from EPFIX (2021–2022) and representative weights of main urban areas at the national level. By household structure, “adults/seniors without children” corresponds to households with more than one person over 18 years old and no children, while “adult/senior with children” corresponds to households with only an adult and children. We used the “margins” post-estimation command in Stata BE 17 to estimate the average marginal effect. Statistical significance levels are indicated as follows: * *p* < 0.1, ** *p* < 0.05, *** *p* < 0.01.

**Table A2:** Full table of descriptive statistics for vegetable purchases.

	Non-Veg Purchasers		Vegetable Purchasers		Difference
	Mean	SD	Mean	SD	
Vegetable portions	0.000	(0.000)	2.490	(2.338)	−2.476 ***
Fruit portions	0.450	(1.256)	2.101	(2.263)	−1.575 ***
Head of Household Characteristics					
Gender of head, 1 = man, 2 = female	1.413	(0.492)	1.431	(0.495)	−0.003
Age of head of household, years	47.609	(15.868)	50.586	(15.409)	−2.350 ***
Education, less than 4 years	0.032	(0.175)	0.036	(0.187)	−0.001
Education, 4–8 years	0.058	(0.234)	0.081	(0.272)	−0.018 **
Education, 8–12 years	0.132	(0.339)	0.156	(0.363)	−0.012
Education, 12–16 years	0.429	(0.495)	0.457	(0.498)	−0.020
Education, more than 16 years	0.348	(0.477)	0.270	(0.444)	0.051 ***
Food Shopper Characteristics					
Gender food shopper, man	0.344	(0.475)	0.156	(0.363)	0.149 ***
Gender food shopper, woman	0.408	(0.492)	0.446	(0.497)	−0.029 *
Gender food shopper, combination	0.247	(0.432)	0.398	(0.489)	−0.120 ***
Age of food shopper, years	47.072	(15.444)	49.585	(14.431)	−1.831 ***
Education, less than 4 years	0.030	(0.171)	0.025	(0.158)	0.007
Education, 4–8 years	0.063	(0.243)	0.081	(0.273)	−0.012
Education, 8–12 years	0.143	(0.350)	0.200	(0.400)	−0.036 ***
Education, 12–16 years	0.447	(0.497)	0.465	(0.499)	−0.019
Education, more than 16 years	0.317	(0.465)	0.228	(0.420)	0.060 ***
Household Composition					
Number of men	1.125	(0.983)	1.569	(1.030)	−0.371 ***
Number of women	1.243	(0.904)	1.489	(0.997)	−0.207 ***
Number of children	0.528	(0.860)	0.703	(0.962)	−0.134 ***
Household Structure					
Single-person	0.348	(0.476)	0.134	(0.340)	0.179 ***
Adults/seniors without children	0.314	(0.464)	0.434	(0.496)	−0.098 ***
An adult/senior with children	0.073	(0.261)	0.052	(0.222)	0.022 ***
Adults/seniors with children	0.265	(0.441)	0.380	(0.485)	−0.102 ***
Household Characteristics					
High income	0.208	(0.406)	0.228	(0.420)	−0.022 *
Observations	2194		12,817		15,011

A portion of FV corresponds to 80 grams. Statistics based on data from EPFIX (2021–2022) and representative weights of main urban areas at the national level. By household structure, “adults/seniors without children” corresponds to households with more than one person over 18 years old and no children, while “adult/senior with children” corresponds to households with only an adult and children. We used the “margins” post-estimation command in Stata BE 17 to estimate the average marginal effect. Statistical significance levels are indicated as follows: * *p* < 0.1, ** *p* < 0.05, *** *p* < 0.01.
